# How Plantar Exteroceptive Efficiency Modulates Postural and Oculomotor Control: Inter-Individual Variability

**DOI:** 10.3389/fnhum.2016.00228

**Published:** 2016-05-13

**Authors:** Arnaud Foisy, Zoï Kapoula

**Affiliations:** IRIS Team, Physiopathologie de la Vision et Motricité Binoculaire, FR3636 Neurosciences Centre National de la Recherche Scientifique University Paris DescartesParis, France

**Keywords:** eye movement, feet cutaneous afferents, inter-individual differences, nociception, plantar quotient, plantar irritating stimulus, postural control, thin plantar insert

## Abstract

In a previous experiment, we showed that among young and healthy subjects, thin plantar inserts improve postural control and modify vergence amplitudes. In this experiment, however, significant inter-individual variability was observed. We hypothesize that its origin could be attributed to a different reliance upon feet cutaneous afferents. In order to test this hypothesis, we re-analyzed the data relative to 31 young (age 25.7 ± 3.8) and healthy subjects who participated in the first experiment after having classified them into two groups depending on their Plantar Quotient (PQ = Surface area of CoP_foam_/Surface area of CoP_firm ground_ × 100). Foam decreases the information arising from the feet, normally resulting in a PQ > 100. Hence, the PQ provides information on the weight of plantar cutaneous afferents used in postural control. Twelve people were Plantar-Independent Subjects, as indicated by a PQ < 100. These individuals did not behave like the Normal Plantar Quotient Subjects: they were almost insensitive to the plantar stimulations in terms of postural control and totally insensitive in terms of oculomotor control. We conclude that the inter-individual variability observed in our first experiment is explained by the subjects' degree of plantar reliance. We propose that plantar independence is a dysfunctional situation revealing inefficiency in plantar cutaneous afferents. The latter could be due to a latent somatosensory dysfunction generating a noise which prevents the CNS from correctly processing and using feet somatosensory afferents both for balance and vergence control: Plantar Irritating Stimulus. Considering the non-noxious nature and prevalence of this phenomenon, these results can be of great interest to researchers and clinicians who attempt to trigger postural or oculomotor responses through mechanical stimulation of the foot sole.

## Introduction

The control of posture involves a multisensory system in which somatosensory information arising from the feet plays an important role (Nashner et al., 1982; Kavounoudias et al., 2001). In their experiment, Kavounoudias et al. (2001) stimulated foot sole mechanoreceptors and ankle tendons muscle spindles with low amplitude mechanical vibrations. They concluded that the regulation of small-amplitude body sways is mainly assigned to feet tactile afferents, whereas ankle muscle proprioception is more involved in the regulation of larger body sways. Consequently, when this source of information is degraded, balance is impaired. For instance, Simoneau et al. (1995) demonstrated an increase of the Surface area of body sway in patients with diabetic neuropathy as compared to healthy subjects. Plantar anesthesia also increases instability both while standing and walking (Fiolkowski et al., 2002). Somatosensory afferents can also be altered experimentally through the interposition of foam between the ground and the subject's feet. All of the experiments resorting to this method have demonstrated that subjects are more unstable when they stand on foam (Chiang and Wu, 1997; Wu and Chiang, 1997; Isableu and Vuillerme, 2006; Patel et al., 2008a,b; Yi and Park, 2009 among others). Indeed, foam decreases the information arising from the feet: Yi and Park (2009) have shown that when healthy subjects are standing on foam, it induces a decrease in plantar cutaneous sensitivity to microfilament touch, similar to the one shown by patients suffering from peripheral sensory neuropathy.

Moreover, an influence of plantar cutaneous afferents upon oculomotor control has been suggested in previous studies and clinical observations. For example, Roll and Roll (1987, 1988) showed that the vibration of extrinsic foot muscles gives rise to an impression of the displacement of a visual target. Erkelens et al. (1989) also showed that vergence accuracy is best during body movements. This led us to investigate the effects of thin plantar inserts on both posture and eye movement control in an ecologic situation during which young and healthy subjects were performing saccade end vergence eye movements while being standing (Foisy et al., 2015). The results of this first experiment showed a decrease in the Surface area and Variance of Speed of the Center Of Pressure (CoP) displacements when the subjects were standing on a bilateral 3-mm-high Medial Arch Support (MAS) or Lateral Arch Support (LAS). These results suggested an increase in stability with corresponding decrease in energy expenditure. Moreover, we also recorded a more posterior position of the CoP with either stimulation compared with the control (no stimulation) condition. Concerning eye movements, the inserts influenced vergence: MAS caused an increase in the phasic amplitude of divergence, and conversely a decrease in the tonic amplitude. In contrast, LAS caused an increase in the tonic amplitude of convergence.

In this experiment, however, we noticed large inter-subject variability both for the postural and oculomotor results. Such inter-individual differences had already been reported elsewhere. As concerns postural control, Patel et al. (2008b) showed that different kinds of foam increased body movements; however, they also mentioned a minority of subjects who did not behave like the others, exhibiting less stablility on firm ground than on foam. Isableu and Vuillerme (2006) also showed that a 2-cm foam surface set beneath the feet of young and healthy subjects increased the instability of the ones who were the most stable on firm ground. Yi and Park (2009) studied the quality of movement detection of subjects standing on a translated platform, either on firm ground or with foam interposition between the feet and the platform. They noticed that the subjects who had the lower detection threshold (i.e., more accurate) on firm ground were the ones who showed the larger increase in that threshold on foam. This means they made greater use of their plantar cutaneous afferents than did the others. As for oculomotor control, Erkelens et al. (1989) studied the accuracy of vergence movements in standing subjects and reported idiosyncratic variations in the dynamics of convergence and divergence. Tyler et al. (2012) assessed the disparity vergence of healthy subjects and also found different subgroups of vergence behavior, most especially with respect to the courses patterns of convergence and divergence dynamics.

However, none of these experiments provided an explanation for the observed results, whether it be in relation to postural or oculomotor control. Given that we used thin plantar inserts, and in accordance with regular clinical observations, we hypothesize that this inter-subject variability might be related to a different reliance upon feet cutaneous afferents. Several methods can be used to test the reliance upon sensory components for the control of balance. For example, the EquiTest Sensory Organisation Test uses computerized dynamic posturography in six conditions, removing one or more sensory inputs involved in postural control to calculate ratios identifying reliance on the different sensory systems (visual, vestibular, somatosensory—see Alahmari et al., 2014). In particular, the somatosensory feedback is altered by an unstable platform. The somatosensory reliance is also classically assessed by comparing the stability (in terms of Surface area of excursions of the Center of Pressure) of the subjects when they stand on firm ground and when they stand on a foam pad. Dujols (1991) called this method “Plantar Quotient” (PQ), Fujimoto et al. (2009, 2012), and Okumura et al. (2015) “foam ratio.” As foam decreases the information arising from the feet (Yi and Park, 2009), foam interposition normally results in a decrease in stability for that condition, revealed by a PQ > 100. Thus, this quotient indicates the weight of plantar cutaneous afferents used in postural control: the higher it is, the more the subject relies on the information arising from his or her feet to keep balance (Oie et al., 2002; Isableu et al., 2011). This method has the advantage of yielding greater antero-posterior and medio-lateral symmetry in the postural responses than the Sensory Organisation Test and is simpler to use in clinical practice (Allum et al., 2002).

Therefore, in the present study, we assessed the somatosensory dependence of the population of our last experiment by means of the Plantar Quotient. Then, we re-analyzed the data of our previous experiment according to each sub-group determined through this quotient. We hypothesize that subjects with PQ < 100, that is, Plantar-Independent Subjects, would be less influenced by the plantar inserts in terms of postural and oculomotor control than Normal Plantar Quotient Subjects (PQ > 100). These questions are important to answer because feet cutaneous afferents are a major source of information for the postural control system (Kavounoudias et al., 2001). A decreased use of this input may provoke instability and could eventually lead to its consequences, such as mechanical pain (Missaoui et al., 2008; Ruhe et al., 2013).

## Materials and methods

### Ethics statement

The investigation adhered to the principles of the Declaration of Helsinki and was approved by the “Comité de Protection des Personnes” (CPP) Ile de France VI (No: 07035), Necker Hospital, in Paris. The subjects gave informed written consent after the nature of the procedure was explained.

### Subjects

Thirty one among the thirty six subjects featured in the previous paper were available to be tested for somatosensory reliance (five were not available to perform the present experiment). They were recruited from paramedical schools, 14 males and 17 females, mean age 25.7 ± 3.8 years, mean height 171.1 ± 8.9 cm, mean body weight 64.9 ± 10.6 kg. Their characteristics are summarized in Table S1.

None of them were taking medication and all of them were asymptomatic. All subjects were emmetropic and wore no glasses. Their visual acuity at close distance was examined by means of Parinaud's reading test. The results were all normal (2 for 29 subjects, 3 for 2 of them). Binocular visual function was also assessed with the stereoacuity TNO test and all values were normal, that is 60″ of arc or lower. They also had an amplitude of accommodation (measured with the push-up method) of 8.99 dioptres (±1.54), which is within Duane's normative data (9.5 ± 2 dioptres; Duane, 1912). The *t*-test test did not show any statistical difference with the theoretical physiologic value (*p* = 0.20).

### Testing conditions, postural, and eye movement recording

In our initial experiment (Foisy et al., 2015), the postural and oculomotor performances of the subjects were recorded using a force platform and an eye-tracker. The following section summarizes the experimental paradigm of that first experiment, the details of which can be found in the original publication.

The force platform was composed of two clogs (produced by TechnoConcept, Céreste, France) whose position was standardized: feet placed side by side, forming a 30° angle with the heels separated by 4 cm. Each clog holds 2 strain gauges (one beneath the metatarsal heads, one beneath the heel) which are force—electric tension transducers. The height and weight of the subjects were factored into the calculations of the CoP displacements. The CoP displacements were recorded over a period of 51.2 s; the equipment contained an Analog—Digital converter of 16 bits and the sampling frequency of the CoP was 40 Hz. The main significantly changing postural parameters were the Surface area of the CoP, its Variance of Speed and its mean antero-posterior (Y) position. The Surface area represents 90% of the instantaneous positions of the CoP included within the confidence ellipse, eliminating the extreme points (Ruhe et al., 2013).

The eye-tracker used to record eye movements was the Chronos Skalar video oculography apparatus, which consists of two infra-red cameras, recording eye movements at a sampling of 200 frames per second. The significantly changing eye movement parameters were the phasic and the tonic amplitudes of convergence and divergence. The phasic amplitude of vergence refers to the amplitude of the movement in its initial stage, which occurs under open-loop control (i.e., without visual feedback). The tonic amplitude corresponds to the second, closed-loop stage of the movement, which is under the influence of visual retroaction.

**Figure 1:**
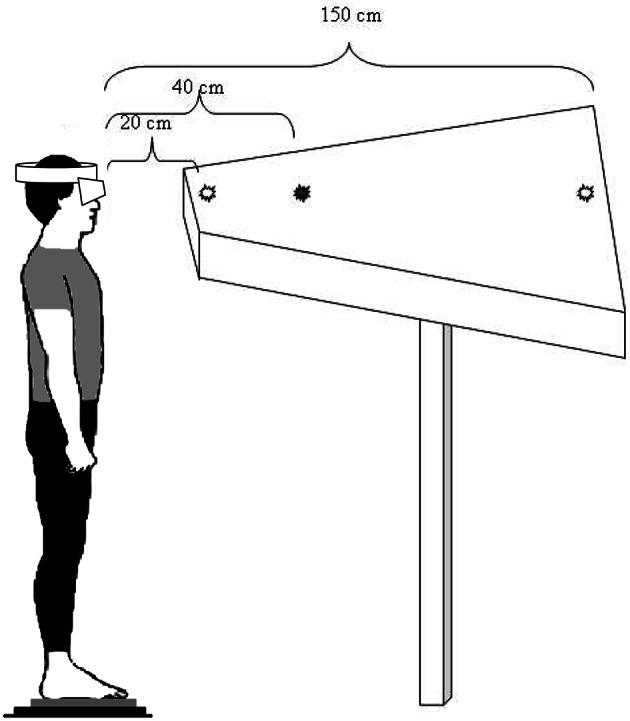
**Visual target and eye movement recording**. The subject is standing on a force platform, staring at the LEDs successively lit, which results in convergence and divergence eye movements.

**Figure 2:**
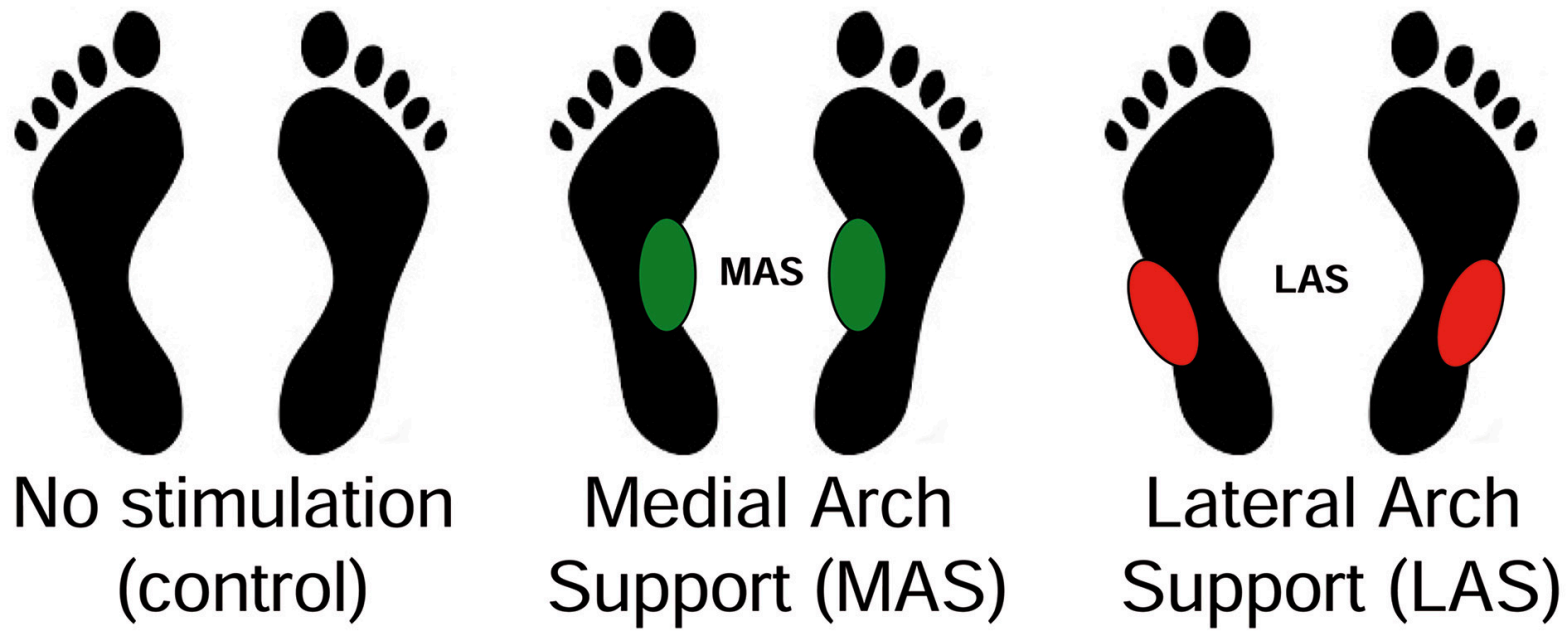
**Posturography testing conditions**. Three millimeter thick inserts (SH 60A, 250 kg/m^3^) were placed beneath the midfoot: on the medial half of the external band of the footprint (MAS condition), or on the lateral half (LAS condition).

The subjects were asked to stand still, barefoot, on the force plate in front of a diode-table set at eye level. They were instructed to fixate on the target LED which lit up, making them do horizontal saccades, 10 convergence and 10 divergence movements, in a random order and overlap paradigm (Figure 1). There were three counterbalanced conditions of plantar stimulation: with no stimulation (Control condition), with a bilateral 3-mm-high Medial Arch Support (MAS condition) and with a bilateral 3-mm-high Lateral Arch Support (LAS condition; Figure 2). These plantar inserts were made of rigid polyester resin, with a shore rating of 60 A and a density of 250 kg/m^3^. A sheet of paper was set beneath the subjects' feet in each condition. In order to place the plantar inserts under the subjects' feet in the right place, Betadine® was brushed under the mid foot of the subjects after they lifted their heel. Then, they placed their heel on the sheet of paper, thus marking it with the Betadine® and lifted it again while the experimenter placed the insert on the medial half of the mark (MAS condition), or on the lateral half of it (LAS condition). This procedure was also done for the control condition, even if no insert was placed under the feet.

We concluded that these plantar inserts improve postural control (decrease of Surface area and Variance of Speed with both inserts) and have influence vergence movements in a distinctive way according to the part of the foot sole being stimulated. For a complete description and explanation of the parameters and results, please see Foisy et al. (2015).

### Somatosensory dependence assessment

We assessed the somatosensory dependence of the subjects using the Plantar Quotient (PQ—Dujols, 1991). The participants were asked to stand still and stare at a target at 90 cm in front of their eyes while standing on the force platform (same device as in the previous experiment). They performed two recordings, one on firm ground and one with a foam surface between their feet and the platform, in a counterbalanced order. The CoP displacements were recorded over a period of 51.2 s and the sampling frequency of the CoP was 40 Hz. We used the same foam as Dujols: Dépron® 6 mm thick, shore 20 A. The PQ for each subject was calculated from these values. This measure is based upon the same principle as the Romberg Quotient (Brandt et al., 1986; Le and Kapoula, 2007) and consists in the ratio between the Surface area of the CoP excursions while the subjects stand on foam and the Surface area while they stand on firm ground: PQ = S_foam_/S_firm ground_ × 100.

We divided our population into two groups: the subjects who showed a normal response, being more stable on firm ground than on foam (PQ > 100), which we will called “Normal Plantar Quotient Subjects” (NPQS); and those who were more stable on foam than on firm ground (PQ < 100), which will be called “Plantar-Independent Subjects” (PIS). To test our hypothesis we re-analyzed the data of our first experiment (Foisy et al., 2015) by comparing the effects of thin plantar inserts on the significantly changing parameters of postural and oculomotor control among the NPQS and in the PIS.

### Statistical analysis

Statistical analysis was performed using Friedman's ANOVA separately on those two groups (procedure of Statsoft/Statistica, release 7.1) since the test of Shapiro–Wilk revealed that some of the distributions were not normal and proved impossible to normalize. *Post hoc* comparisons were done whenever necessary using the test of Wilcoxon, with *p* < 0.05 considered as significant. The magnitudes of the differences were assessed by the effect size (Cohen's d).

## Results

### Plantar Quotient results and group constitution

We obtained quite a similar mean PQ (126) as Dujols did on 69 normal subjects (132). Nineteen subjects had a PQ higher than 100 (mean PQ = 162 ± 61) and were categorized as NPQS. Twelve subjects had a PQ below 100 (mean PQ = 69 ± 23) and were categorized as PIS.

Mann-Whitney *U*-tests showed that the two groups did not have significantly different ages (*z* = 0.16, *p* = 0.87), heights (*z* = −0.85, *p* = 0.39), weights (*z* = −0.16, *p* = 0.87), stereoacuity (*z* = 1.46, *p* = 0.11), visual acuity (*z* = −0.49, *p* = 0.25), and amplitude of accommodation (*z* = −0.16, *p* = 0.87). The only significantly difference between the two groups was their PQ (*z* = −4.62, *p* < 0.01, *d* = 2.02), the PIS having a lower Plantar Quotient than the NPQS due to a higher Surface area on firm ground (*z* = 2.43, *p* = 0.01, *d* = 3.05; see Table S1).

### Comparison of the effects of thin plantar inserts within each group

#### Postural control

**Figure 3:**
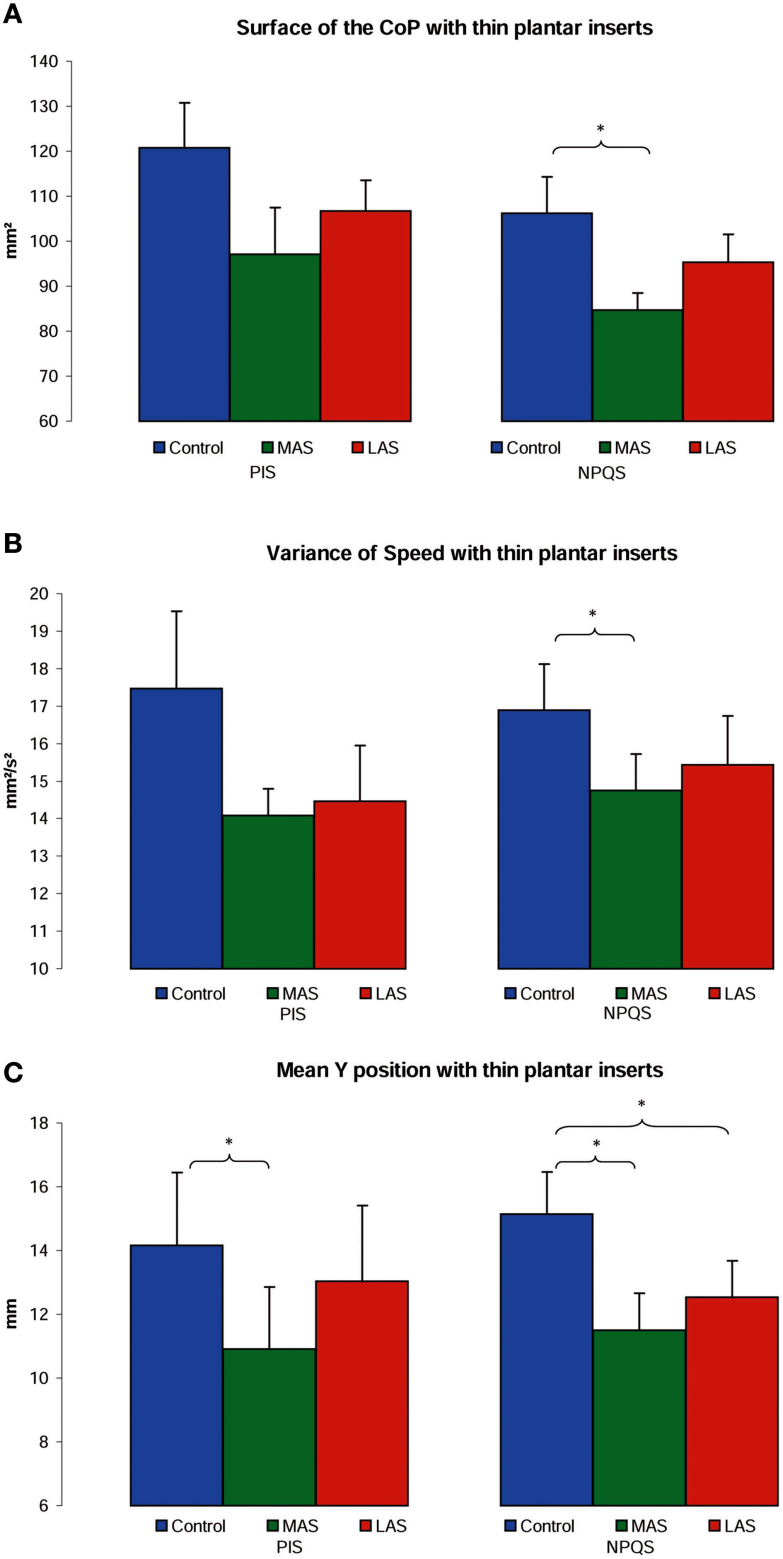
**Postural performances among PIS and NPQS**. Mean of the Surface area **(A)**, Variance of Speed **(B)**, and Y position **(C)** of the Center of Pressure excursions for each testing condition. Error bars represent the standard error. Asterisks indicate significant differences, with ^*^p < 0.05.

For the NPQS, the ANOVA showed a main effect of the sole stimulation conditions on the Surface area [$\chi_{\left(2,\;19\right)}^{2}$ = 9.58, *p* = 0.01]. The test of Wilcoxon showed that the Surface area was statistically lower with MAS than in Control condition (*z* = 2.25, *p* = 0.02, *d* = 0.78). A borderline difference between Control and LAS (*z* = 1.81, *p* = 0.07, *d* = 0.34) and between MAS and LAS was also observed (*z* = 1.85, *p* = 0.06, *d* = 0.47; Figure 3A).

Concerning the Variance of speed, there was a main effect of the sole stimulation conditions [$\chi_{\left(2,\;19\right)}^{2}$ = 9.58, *p* = 0.01], with a lower Variance of Speed with MAS than in Control condition (*z* = 2.50, *p* = 0.01, *d* = 0.44; Figure 3B).

Another Friedman's ANOVA showed a main effect of the stimulations on the antero-posterior (Y) position of CoP [$\chi_{\left(2,\;19\right)}^{2}$ = 6.00, *p* = 0.05]. The Y position proved more posterior with MAS than on firm ground (*z* = 2.54, *p* = 0.01, *d* = 0.68), and with LAS than on firm ground (*z* = 2.09, *p* = 0.04, *d* = 0.49; Figure 3C).

For the PIS, there was only a main effect of the stimulation conditions on the antero-posterior (Y) position of CoP [$\chi_{\left(2,\;12\right)}^{2}$ = 9.50, *p* = 0.01]. The Y position was more posterior with MAS than on firm ground (*z* = 2.98, *p* < 0.01, *d* = 0.44; Figure 3C).

On the whole, we observed more statistical effects with the plantar inserts among the NPQS. The only significant effect among the PIS was on the antero-posterior (Y) position of CoP and only with MAS. The results are summarized in Figures 3A–C and Table 1.

**Table 1:** **Postural performances of the subjects (during the initial experiment)**.

	**Control**	**MAS**	**LAS**
**SURFACE AREA OF THE COP (mm^2^**			
PIS	121 ± 10 [99, 143]	97 ± 10 [74, 120]	107 ± 7 [92, 122]
NPQS	106 ± 8 [89, 123]	85 ± 4 [77, 93]	95 ± 6 [82, 109]
**VARIANCE OF SPEED OF THE CoP (mm^2^/s^2^)**			
PIS	17 ± 2 [13, 22]	14 ± 1 [13, 16]	14 ± 1 [11, 17]
NPQS	17 ± 1 [14, 19]	15 ± 1 [13, 17]	15 ± 1 [13, 18]
**MEAN Y POSITION OF THE CoP (mm)**			
PIS	14 ± 2 [9, 19]	11 ± 2 [7, 15]	13 ± 2 [8, 18]
NPQS	15 ± 6 [12, 18]	11 ± 1 [9, 14]	13 ± 1 [10, 15]

*Means, standard errors and 95% Confidence Intervals of postural parameters for each condition*.

#### Oculomotor control

**Figure 4:**
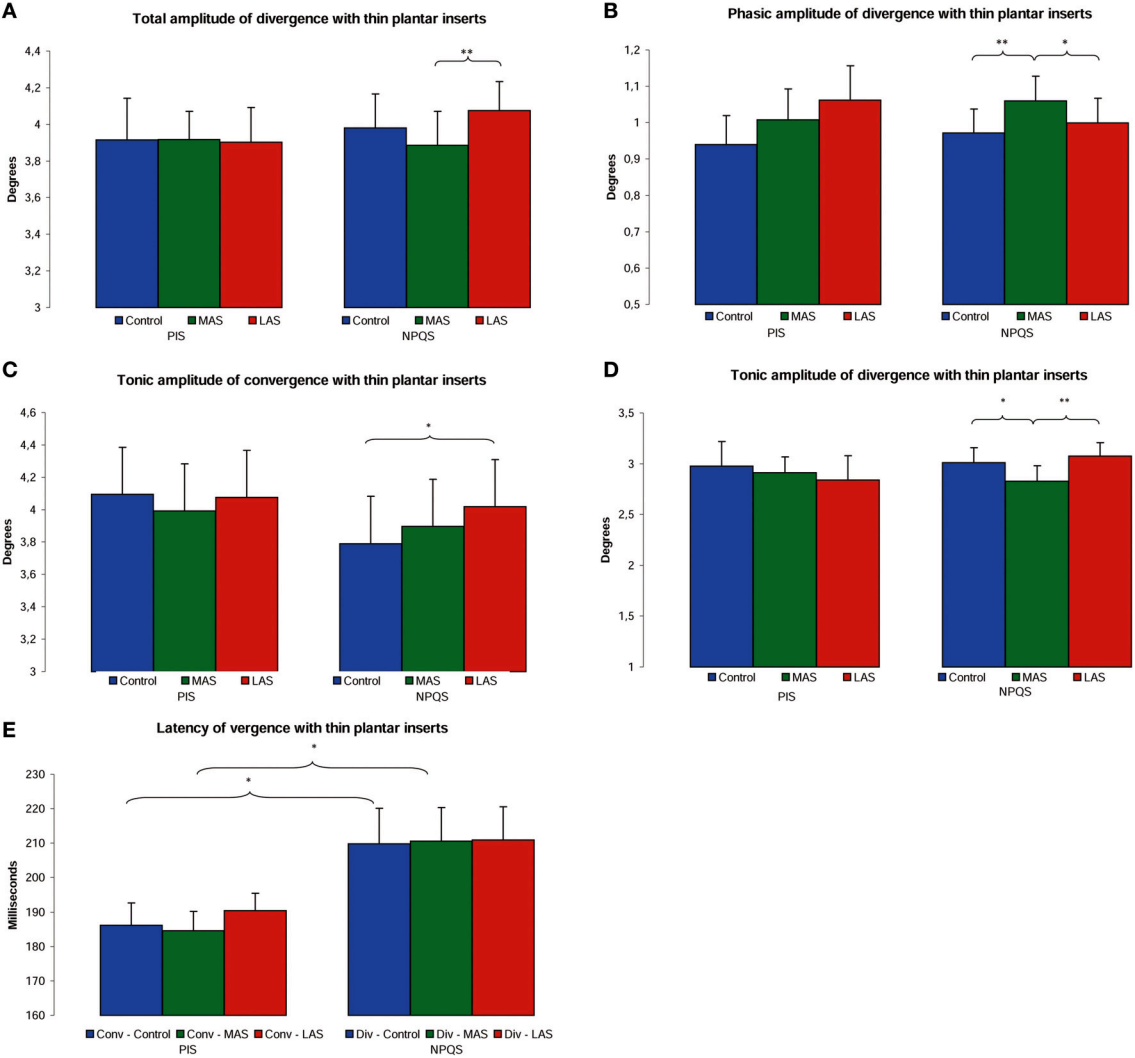
**Oculomotor performances among PIS and NPQS**. Mean of the total amplitude of divergence **(A)**, phasic amplitude of divergence **(B)**, tonic amplitude of convergence **(C)**, and divergence **(D)**, and latency of convergence and divergence **(E)** for each testing condition. Error bars represent the standard error. Asterisks indicate significant differences, with ^*^p < 0.05; ^**^p < 0.01.

For the NPQS, the ANOVA showed a main effect of the stimulation conditions on the total amplitude of vergence [$\chi_{\left(5,\;19\right)}^{2}$ = 44.71, *p* < 0.01]. The test of Wilcoxon showed a statistically higher amplitude of divergence with LAS than MAS (*z* = 2.98, *p* < 0.01, *d* = 0.25; Figure 4A).

As regards the phasic amplitude of vergence, the ANOVA also showed a main effect [$\chi_{\left(5,\;19\right)}^{2}$ = 48.86, *p* < 0.01]. The MAS induced an increase of the phasic amplitude of divergence as compared to the Control condition (*z* = 2.78, *p* = 0.01, *d* = 0.31) and to LAS (*z* = 2.41, *p* = 0.02, *d* = 0.20; Figure 4B).

Concerning the tonic amplitude of vergence, there was a main effect of the sole stimulation conditions [**χ**_(5, 19)_ = 39.54, *p* < 0.01]. MAS induced a decrease of the tonic amplitude of divergence as compared to the Control condition (*z* = 2.45, *p* = 0.01, *d* = 0.27) and to LAS (*z* = 3.10, *p* < 0.01, *d* = 0.40). There was also a significant increase of the tonic amplitude of convergence with LAS as compared to the Control condition (*z* = 2.25, *p* = 0.02, *d* = 0.18; Figures 4C,D).

Regarding the latencies of convergence and divergence, the Friedman's ANOVA also showed main effects of the sole stimulation conditions [**χ**_(5, 19)_ = 12.20, *p* = 0.03]. Divergence had a statistically significant higher latency than convergence in the Control condition (*z* = 2.21, *p* = 0.03, *d* = 0.64) and in MAS condition (*z* = 2.21, *p* = 0.03, *d* = 0.76; Figure 4E).

The PIS displayed no significant differences relative to the total, phasic and tonic amplitudes, nor were any differences observed in relation to the latencies of vergence. The results are summarized in Figures 4A–E and Table 2.

**Table 2:** **Oculomotor performances of the subjects (during the initial experiment)**.

	**Control**	**MAS**	**LAS**
**DIVERGENCE**			
**Total amplitude (degrees)**			
PIS	3.91±0.23 [3.41, 4.42]	3.92±0.15 [3.58, 4.25]	3.90±0.19 [3.48, 4.32]
NPQS	3.98±0.19 [3.59, 4.37]	3.89±0.18 [3.50, 4.27]	4.08±0.16 [3.74, 4.41]
**Phasic amplitude (degrees)**			
PIS	0.99±0.05 [0.76, 1.12]	1.06±0.05 [0.82, 1.19]	1.05±0.05 [0.85, 1.27]
NPQS	0.97±0.07 [0.83, 1.11]	1.06±0.07 [0.92, 1.20]	1.00±0.07 [0.86, 1.14]
**Tonic amplitude (degrees)**			
PIS	2.97±0.24 [2.44, 3.51]	2.91±0.16 [2.56, 3.26]	2.84±0.24 [2.32, 3.36]
NPQS	3.01±0.15 [2.69, 3.32]	2.83±0.15 [2.50, 3.15]	3.08±0.13 [2.80, 3.35]
**Latency (milliseconds)**			
PIS	206±8 [188, 225]	215±8 [196, 233]	217±10 [194, 239]
NPQS	210±10 [188, 232]	211±10 [190, 231]	211±10 [191, 231]
**CONVERGENCE**			
**Total amplitude (degrees)**			
PIS	5.59±0.37 [4.78, 6.40]	5.56±0.37 [4.74, 6.38]	5.61±0.39 [4.76, 6.47]
NPQS	5.44±0.43 [4.53, 6.35]	5.60±0.41 [4.74, 6.46]	5.66±0.37 [4.89, 6.44]
**Phasic amplitude (degrees)**			
PIS	1.49±0.14 [1.18, 1.81]	1.57±0.16 [1.21, 1.92]	1.54±0.17 [1.17, 1.91]
NPQS	1.81±0.16 [1.47, 2.14]	1.70±0.15 [1.39, 2.02]	1.65±0.13 [1.38, 1.91]
**Tonic amplitude (degrees)**			
PIS	4.09±0.29 [3.45, 4.74]	3.99±0.29 [3.34, 4.64]	4.07±0.29 [3.43, 4.72]
NPQS	3.79±0.29 [3.18, 4.40]	3.90±0.29 [3.29, 4.51]	4.02±0.29 [3.41, 4.63]
**Latency (milliseconds)**			
PIS	190±6 [176, 203]	190±6 [177, 202]	193±7 [178, 208]
NPQS	186±6 [173, 200]	185±6 [173, 196]	190±8 [173, 208]

*Means, standard errors and 95% Confidence Intervals of oculomotor parameters for each condition*.

## Discussion

The result of that experiment is that the PIS are far less sensitive than NPQS to thin plantar inserts in relation to both their postural and oculomotor control, thus confirming our hypothesis.

### Plantar Quotient results

With a mean PQ of 126 our results are in line with the literature (Dujols, 1991) and confirm that people are more stable on firm ground than on foam (Chiang and Wu, 1997; Wu and Chiang, 1997; Patel et al., 2008a,b)—a result that can be expressed in terms of a PQ higher than 100. It is worth noting that in the literature the authors use thick (several cm) and compliant foam support surfaces that lead both to biomechanicala (Patel et al., 2008a,b; Yi and Park, 2009) and sensorial effects, the latter involving simultaneous plantar exteroception and proprioception (Chiang and Wu, 1997; Wu and Chiang, 1997; Patel et al., 2008a,b). Here we used thin and firm foam in order to focus the action on plantar cutaneous afferents (following Dujols, 1991; Leporck and Villeneuve, 1996).

### Comparison of the effects of thin plantar inserts within each group

The PIS did not show any significant change in the parameters of postural control whereas the NPQS still did. The only persisting postural effect of the inserts among the PIS is a backward shift of the mean (Y) position of the CoP, and only with MAS compared to Control. By contrast, both MAS and LAS produce that effect on the NPQS. Interestingly, in absolute terms, the Surface area changed to a greater degree in the PIS group than among the NPQS, most especially with MAS compared to Control. Yet, variance was higher in the PIS, leading to lack of statistically significant effect.

Regarding eye movements, the difference between the PIS and the NPQS is even more obvious. As was observed in relation to the postural results, the two groups did not behave the same: the vergence movements of the PIS are not affected at all by the plantar stimulations, contrary to the ones of the NPQS. As concerns the latencies of convergence and divergence, for the NPQS the latencies of divergence were longer than the latencies of convergence in Control and MAS conditions. We suggested (Foisy et al., 2015) that this unusual result could be explained by the fact that, when standing, the control of balance is more difficult when one fixates a distant target (i.e., divergence) than at close distance (i.e., convergence; Kapoula and Le, 2006; Le and Kapoula, 2007), which implies a decrease in the use of visual and oculomotor cues in favor of somatosensory afferents (Le and Kapoula, 2007). Hence, our observation according to which sensory re-weighting could explain the need for longer processing times for divergence than for convergence. This idea is supported by the fact that the PIS do not show any difference in their convergence and divergence latencies with any stimulation. It seems therefore that these subjects cannot use their plantar cutaneous afferents to compensate for the decrease of the visual cues.

These results confirm our hypothesis: among PIS, the effects of thin plantar inserts on postural control almost entirely disappear and are totally absent in relation to oculomotor control. In contrast, these effects persist among NPQS.

### Plantar independence or plantar inefficiency?

Plantar cutaneous afferents normally represent very important cues that the CNS uses to ensure balance during quiet stance (Kavounoudias et al., 2001). Hence, a decrease of their use (especially among healthy subjects) does not seem to serve any physiological purpose. It is therefore more likely that the PIS are victim of a latent plantar somatosensory dysfunction. As foam interposition between ground and feet acts as a plantar anesthesia (Yi and Park, 2009), its impairing consequences on postural control appear as a physiological situation. On the contrary, the improvement of balance on foam (i.e., Plantar Independence) could be due to a dysfunction of the sole receptors, which is suppressed when the subject stands on foam.

None of the studies reporting idiosyncrasies in vergence or postural control mentioned in the introduction proposed any explanation for these observations. We propose that the presence, among the PIS of a non-noxious plantar somatosensory dysfunction could explain our results. Clinicians described a pathophysiological entity called “Plantar Irritating Stimulus” (Janin, 2009; Marc et al., 2015). Leporck and Villeneuve (1996) were the first to mention them, defining them as “conscious or unconscious podal nociceptive zones that provoke a change of posture or balance in standing humans.” As the neural basis of that entity remains unknown (either mechanoreceptors and Aβ fibers, or nociceptors and Aδ/C fibers, or both), the generic term “irritating stimulus” is preferred to “nociception,” often related to a felt pain which uses the spinothalamic tract of nociception. The word “nociceptive” was initially proposed after the definition of Sherrington (1910), who considered nociception as “a sensorial stimulation of high intensity that is able to harm the integrity of the organism.”

Janin (2009) suggested that an increase of pressure beneath certain plantar zones (like the first metatarsal head) could entail an increase of the frequency discharge of the sole receptors (after Vedel and Roll, 1982; Ribot-Ciscar et al., 1989). The latter would constitute a “noise” which could be assimilated to an unconscious nociception and yield potentially harmful effects such as instability. It is now well-known that even unconscious plantar stimuli can modify postural performances during quiet stance through a stochastic resonance phenomenon (Priplata et al., 2002, 2003, 2006). The latter classically refers to a situation in which the addition of a noise (i.e., subliminal stimulus) to an input of a nonlinear system can improve its sensitivity (see Fallon and Morgan, 2005). Yet, the addition of one such noise can produce either beneficial or detrimental sensorimotor effects. Collins et al. (1997) found that the addition of noise enhances the ability to perceive sub-threshold tactile stimuli but degrades the perception of supra-threshold tactile stimuli. In our experiment, the plantar inserts correspond to the latter scenario as they were indeed felt by the subjects. Furthermore, Fallon and Morgan (2005) showed that tactile detection implies a tuneable stochastic resonance phenomenon. In this form of stochastic resonance the importance of the noise which is added to an input must be adapted to an appropriate frequency of body sway in order to improve the detection of a narrow range of body sway frequencies by the postural control system and produce optimized responses for these frequencies. For example, Ribot-Ciscar et al. (2013) showed that the ability to detect the direction of sub-threshold movements of the ankle significantly increases when a low-magnitude mechanical noise (20 or 30 μm vibrations) is applied to ankle tendons, but significantly decreases for high-magnitude noises (100 or 280 μm vibrations). Thus, a calibrated extrinsic noise which is experimentally added can more easily match the receptors' optimal threshold and therefore have positive sensorimotor effects. On the contrary, in case of Plantar Irritating Stimulus, the intrinsic source of noise (i.e., increase of the frequency discharge of the receptors) depends on plantar pressure distribution and is not controlled. Hence, such a noise can easily exceed the receptors' threshold and lead to a difficulty of integration of the increased plantar afferents (Weerakkody et al., 2003). Thus, following this rationale, the PIS would not have a lack of plantar afferents but an excess. Confronted with these maladjusted signals, the CNS would neglect them, as our results suggest. Foam seems to act like a filter, smoothing plantar pressure distribution (Chiang and Wu, 1997; Wu and Chiang, 1997) and hence decreasing the excessive and disruptive plantar signals, as suggests the increase of stability of PIS when they stand on the foam pad.

Leporck and Villeneuve (1996) originally proposed that Plantar Irritating Stimuli could be detected either by the improvement of the quality of the movement of a clinical test, or by the improvement of stability upon posturographic parameters when the affected subjects stand on foam. Marc et al. (2015) used the clinical method to detect Plantar Irritating Stimuli and then assessed their stability with a force platform. He also found that the subjects affected by Plantar Irritating Stimuli had a greater Surface area of CoP on firm ground than on foam, and conversely for the unaffected subjects. Our results are in agreement with his, which suggests that both methods are able to detect Plantar Irritating Stimuli and confirms the proposition of Leporck and Villeneuve (1996). Furthermore, as we here show that the influence of thin plantar inserts almost completely vanishes among PIS, it suggests that the presence of Plantar Irritating Stimulus prevents those subjects to properly use their plantar cutaneous afferents, both for postural and oculomotor control.

The remaining effect of MAS upon the antero-posterior position of the CoP among the PIS suggests that this dysfunction affects the fast adaptating mechanoreceptors (FA) more so than the slow adaptating mechanoreceptors (SA). Indeed, the first ones are particularly useful at signaling of changes in the direction of movement (Yi and Park, 2009), speed and acceleration (Jeka et al., 2004), while the second rather serve to provide information on the importance of constant pressure (Burgess and Perl, 1973; Esteky and Schwark, 1994). Moreover, it is known that a medial plantar stimulation entails a supination of the feet (see Foisy et al., 2015) and therefore decreases the excess of pressure beneath the first metatarsal head which is considered to be responsible for the Plantar Irritating Stimulus. Hence, this can also account for the remaining effect on the Y position of the CoP and the more important variance observed with MAS in the PIS group.

## Conclusion

The interindividual variability in the results of our first study (Foisy et al., 2015) can be explained by the degree to which subjects make use of plantar afferents. Furthermore, as it is correlated with an inability to properly use plantar cutaneous afferents, the situation of Plantar-Independence rather appears as a plantar exteroceptive inefficiency. It seems to be related to the non-physiological phenomenon of Plantar Irritating Stimulus; however, further research is required to objectify that clinical entity and its neural correlates. These results are an invitation to researchers and clinicians to take into account the inter-individual differences concerning the use of the plantar afferents when dealing with standing subjects.

## Author contributions

Conceived and designed the experiment/Acquisition and analysis of data/Interpretation of data: AF, ZK. Drafting/revising the article: AF, ZK. Final approval of the version to be submitted: AF, ZK. Agreement to be accountable for all aspects of the work: AF, ZK.

### Conflict of interest statement

The authors declare that the research was conducted in the absence of any commercial or financial relationships that could be construed as a potential conflict of interest.

## Abbreviations

CoP: Center of Pressure
MAS: Medial Arch Support
LAS: Lateral Arch Support
NPQS: Normal Plantar Quotient Subjects
PIS: Plantar-Independent Subjects
PQ: Plantar Quotient.
