# Supplementary material for: How Plantar Exteroceptive Efficiency Modulates Postural and Oculomotor Control: Inter-Individual Variability
Source: Front Hum Neurosci. 2016 May 13;10:228. doi: 10.3389/fnhum.2016.00228 (PMC4866577; doi:10.3389/fnhum.2016.00228)
Supplement: Supplementary file 1 [file Table1.DOC]

Table S1

*Subject’s characteristics and postural performances during PQ assessment*

For each subject: Plantar Quotient (PQ), Height (cm), Weight (kg), stereoacuity (TNO), visual acuity at close distance (Parinaud, mean of both eyes) and postural performances (Surface area of CoP in mm²) measured for PQ assessment. Normal values are <100 for TNO, <3 for Parinaud and 9.5 ± 2 for the amplitude of accommodation.

| Subject | Group | PQ | Gender | Age | Height  (cm) | Weight  (kg) | TNO | Parinaud | Accom-modation | Surface area on firm ground | Surface area on foam |
| --- | --- | --- | --- | --- | --- | --- | --- | --- | --- | --- | --- |
| S3 | PIS | 93 | F | 33 | 163 | 51 | 30 | 2 | 6,25 | 100 | 93 |
| S6 | PIS | 86 | F | 22 | 172 | 66 | 60 | 2 | 11,11 | 61 | 53 |
| S7 | PIS | 94 | F | 22 | 156 | 58 | 30 | 2 | 8,96 | 153 | 143 |
| S11 | PIS | 41 | M | 25 | 175 | 72 | 60 | 2 | 7,23 | 296 | 121 |
| S13 | PIS | 81 | F | 23 | 170 | 57 | 30 | 2 | 10,20 | 70 | 57 |
| S18 | PIS | 22 | M | 24 | 182 | 72 | 30 | 2 | 8,70 | 290 | 63 |
| S21 | PIS | 91 | F | 24 | 167 | 85 | 60 | 2 | 6,65 | 204 | 186 |
| S25 | PIS | 63 | F | 25 | 158 | 53 | 60 | 2 | 8,85 | 254 | 159 |
| S27 | PIS | 76 | M | 30 | 186 | 72 | 60 | 2 | 10,53 | 180 | 137 |
| S28 | PIS | 76 | F | 26 | 158 | 55 | 30 | 2 | 7,21 | 125 | 94 |
| S29 | PIS | 59 | F | 29 | 168 | 61 | 30 | 2 | 11,32 | 184 | 109 |
| S31 | PIS | 49 | M | 24 | 176 | 74 | 60 | 2 | 10,53 | 122 | 59 |
| *M* |  | 69 |  | 25,6 | 169,3 | 64,7 | 45,00 | 2,00 | 8,96 | 170 | 106 |
| *SD* |  | 23 |  | 3,4 | 9,6 | 10,4 | 15,67 | 0,00 | 1,80 | 23 | 13 |
| p values for conditions | |  |  |  |  |  |  |  |  | 0,002 | |
| S1 | NPQS | 115 | F | 31 | 172 | 74 | 30 | 2 | 8,33 | 98 | 112 |
| S2 | NPQS | 124 | M | 25 | 173 | 71 | 30 | 2 | 12,05 | 93 | 115 |
| S4 | NPQS | 109 | F | 22 | 168 | 62 | 60 | 2 | 8,88 | 356 | 388 |
| S5 | NPQS | 255 | M | 23 | 184 | 75 | 60 | 2 | 9,32 | 69 | 175 |
| S8 | NPQS | 346 | M | 31 | 185 | 67 | 60 | 2 | 7,79 | 52 | 181 |
| S9 | NPQS | 158 | M | 22 | 183 | 76 | 60 | 2 | 10,71 | 84 | 132 |
| S10 | NPQS | 163 | F | 35 | 160 | 61 | 30 | 2 | 6,67 | 143 | 233 |
| S12 | NPQS | 120 | F | 22 | 169 | 52 | 30 | 2,5 | 9,55 | 212 | 254 |
| S14 | NPQS | 114 | M | 24 | 173 | 76 | 30 | 2 | 9,52 | 52 | 59 |
| S15 | NPQS | 229 | M | 26 | 178 | 79 | 60 | 2 | 10,71 | 94 | 215 |
| S16 | NPQS | 173 | M | 33 | 187 | 89 | 15 | 2 | 9,38 | 115 | 198 |
| S17 | NPQS | 114 | F | 23 | 171 | 57 | 30 | 2 | 9,23 | 65 | 74 |
| S19 | NPQS | 130 | F | 22 | 162 | 52 | 30 | 2 | 7,79 | 169 | 220 |
| S20 | NPQS | 166 | M | 23 | 166 | 54 | 60 | 2 | 7,41 | 94 | 155 |
| S22 | NPQS | 207 | F | 25 | 168 | 55 | 15 | 2 | 7,14 | 96 | 199 |
| S23 | NPQS | 137 | F | 23 | 168 | 51 | 15 | 2 | 8,15 | 35 | 48 |
| S24 | NPQS | 153 | F | 25 | 160 | 57 | 15 | 2 | 8,20 | 51 | 79 |
| S26 | NPQS | 164 | M | 29 | 166 | 64 | 15 | 2 | 9,52 | 27 | 45 |
| S30 | NPQS | 109 | M | 25 | 180 | 64 | 30 | 3 | 10,71 | 139 | 151 |
| *M* |  | 162 |  | 25,7 | 172,3 | 65,1 | 35,53 | 2,08 | 9,00 | 107 | 160 |
| *SD* |  | 61 |  | 4,1 | 8,4 | 10,9 | 18,17 | 0,25 | 1,40 | 17 | 20 |
| p values for conditions | |  |  |  |  |  |  |  |  | 0,000 | |
| p values for groups | | 0,000 |  | 0,870 | 0,394 | 0,871 | 0,113 | 0,253 | 0,871 | 0,015 | 0,074 |
